# Development and evaluation of the low back pain questionnaire for personnel working on islands

**DOI:** 10.3389/fpubh.2025.1655747

**Published:** 2025-08-19

**Authors:** Yongbin Feng, Wenyu Tang, Yajun Cheng, Xiaoyi Zhou, Xianzhao Wei

**Affiliations:** ^1^Department of Spinal Surgery, Changhai Hospital, Shanghai, China; ^2^School of Nursing, Beihua University, Jilin, China

**Keywords:** low back pain, questionnaire development, personnel working on islands, reliability, validity, biopsychosocial model

## Abstract

**Background:**

Low back pain (LBP) is a significant musculoskeletal disorder with multifactorial causes, affecting workforce productivity globally. Personnel working on islands face heightened LBP risk due to intensive training (e.g., heavy lifting, prolonged standing) and harsh environmental conditions (high temperature, humidity, solar radiation). Existing LBP questionnaires, such as the Roland-Morris Disability Questionnaire and Oswestry Disability Index, lack specificity for island personnel's unique challenges. This study aimed to develop and validate the Low Back Pain Questionnaire (LBPQ) tailored to this population, aligning with the biopsychosocial medical model.

**Methods:**

The LBPQ development followed a six-step process: conceptual definition, item generation, purification, dimension extraction, and reliability/validity testing. Through literature review, expert discussions, and interviews with 30 personnel working on islands, a 50-item initial scale was refined to 25 items across five dimensions: Pain Severity, Training, Daily Life, Psychological Impact, and Island Specificity. The final scale was administered to 600 personnel working on islands (experimental group) and 600 personnel working on land (control group). Reliability was assessed via Cronbach's α, while validity included factor analysis, convergent/discriminant validity tests, and correlation with the Visual Analog Scale (VAS) and Oswestry Disability Index (ODI).

**Results:**

The LBPQ demonstrated excellent reliability for personnel working on islands (Cronbach's α = 0.978) and good validity, with a KMO value of 0.967 and a cumulative variance contribution rate of 93.322%. Confirmatory factor analysis showed optimal model fit (χ^2^/d*f* = 1.354, CFI = 0.997, RMSEA = 0.024). Convergent and discriminant validity were superior in the island group compared to the control group. Strong correlations were observed with VAS (*r* = 0.675) and ODI (*r* = 0.824), confirming alignment with established scales.

**Conclusion:**

The LBPQ is a reliable and valid tool for assessing LBP in personnel working on islands, addressing their unique environmental and occupational risks. It enhances clinical understanding of LBP severity and psychosocial impacts, enabling targeted prevention and intervention strategies. Future research should validate its applicability across diverse island environments and further refine its brevity.

## Background

Low back pain (LBP) has been a prevalent healthcare problem in western countries for many years, increasing recently. Previous studies have revealed that LBP is a multifactorial musculoskeletal disorder. Environment, occupation, demography, health behavior and sociopsychology have been identified as risk factors associated with LBP (1–5). LBP is largely responsible for the decline of the labor force, causing an ~$28 billion loss in the United States (6).

As a special group, personnel working on islands are at high risk of experiencing LBP because of the demands of their jobs. Army-related trainings, which may include heavy lifting, long-time standing at attention, and excessive pushing/pulling/twisting/bending, are closely associated with LBP occurrence (7, 8). In previous reports, LBP is shown to be a common reason for individuals to seek medical consultation (9). The prevalence of LBP in the land force ranges from 61% to 84% in different countries (9, 10). Increased prevalence of LBP limits the training efficiency. The island environment is known for its high temperature, high humidity, high salt content, high solar radiation and long periods of sunshine (11). Such a harsh environment increases LBP morbidity. Previous studies have demonstrated that musculoskeletal disorder is the most frequently-occurring symptom of personnel working on islands (12). Since our understanding of health has shifted from a biomedical model to a biopsychosocial medical model in recent years (13), it is important for clinicians to emphasize not only diagnosis and treatment, but also the social and mental health of personnel.

Several self-reported questionnaires have been developed to evaluate LBP status, including the Roland-Morris disability questionnaire (14), Oswestry disability index (15), Quebec back pain disability scale (16), and many other scales (17), some of which have been adapted into a simplified Chinese version for LBP evaluation (18–23). However, none of these questionnaires was developed specifically to evaluate individuals with LBP in such a special environment among such a special group. Therefore, this study was designed to develop a low back pain questionnaire (LBPQ) that conforms to the characteristics of personnel working on islands as well as to evaluate the relevant measurement properties. Having an accurate and targeted evaluation may help clinicians better diagnose, deal with and prevent LBP in personnel working on islands. This specific tool would provide better physical and medical readiness.

The LBPQ is the first questionnaire specifically targeting personnel working on islands in order to better understanding the severity and status of backpain in these island personnel. The objective of the present study was to develop a self-reported LBPQ for personnel working on islands, following the standard procedure for questionnaire development, and ensuring good reliability and validity.

## Materials and methods

### Design and development of the LBPQ

The LBPQ development process adhered to standard procedures for instrument development, comprising six parts: conceptual definition, initial item establishment, item purification, dimension generation, reliability and validity testing. Experts conducted field investigations on islands to understand environmental and climatic characteristics. Through individual interviews and group discussions with personnel working on islands, experts gained insights into the incidence of lumbago and leg pain, daily work and living conditions, and training activities. Literature review, book research, and expert panel discussions were employed to define relevant concepts and explore the impact of pain severity and frequency on training, island life, and psychological wellbeing. Following extensive discussion, five dimensions were initially identified: “Pain Severity,” “Training,” “Daily Life,” “Psychological Impact,” and “Island Specificity,” resulting in a pool of 50 items with satisfactory psychometric properties. After pre-testing with 30 randomly selected personnel working on islands, revisions were made based on feedback, culminating in a final 25-item scale using a Likert 5-point scale for scoring.

### Participants

Among personnel working on islands, those who had lumbago and leg pain as the main complaint were randomly selected as the experimental group, while personnel working on land with lumbago and leg pain as their main complaint were selected as the control group. Those who had working time < 3 months; history of spinal surgery or trauma; history of rheumatic diseases; or who refused to participate in the survey were excluded. Prior to the official survey, the purpose and process of the study was clearly explained to each participant, including demonstrating the questionnaire instructions and examination methods, so that they fully understood the purpose and significance of the study.

### Sample size calculation

Under the condition of simple random sampling, the expert panel adopted the formula for investigating the sample size: *N* = *Z*^2^σ^2^/*d*^2^, where *N* represents the required sample size for the research; *Z* is the statistical variable for the confidence level. In this study, a 95% confidence level was taken, so the statistical variable *Z* was 1.96; σ was the population standard deviation, which was taken as 0.5 in this study; d was half of the confidence interval, i.e., the allowable error or survey error. In this study, the sampling error was limited to no more than 4%. After calculation, *N* = 600.

### Statistical analysis

The LBPQ for personnel working on islands was scored and statistically analyzed. Each item was scored as 1, 2, 3, 4, or 5, with a total score ranging from 25 to 125. If any item was left blank or multiple choices were selected, the LBPQ was considered invalid and not included in the statistics. After summarizing the data, reliability and validity analyses were performed. Reliability analysis was assessed through internal consistency of the LBPQ, while validity analysis was completed through exploratory and confirmatory factor analyses. Statistical analysis was performed using AMOS 24.0 and SPSS 22.0 software. Measurement data with normal distribution were expressed as mean ± standard deviation, while non-normally distributed data were expressed as count or percentage.

### Reliability analysis

Internal consistency of the LBPQ was assessed by calculating Cronbach's alpha coefficient to evaluate reliability. When the alpha coefficient for the overall questionnaire was ≥0.60, and the alpha coefficient for each dimension was ≥0.60, the results were considered satisfactory.

### Validity analysis

Validity analysis was conducted through exploratory factor analysis, confirmatory factor analysis, KMO test, Bartlett's test of sphericity, convergent validity test, discriminant validity test, model fit, and Pearson correlation analysis. The KMO test and Bartlett's test of sphericity were used to determine whether the KMO value and Bartlett's value of the LBPQ were higher than 0.8 to determine whether the LBPQ was suitable for factor analysis. In convergent validity, discriminant validity, and model fit, relevant tests were conducted separately for personnel working on land and personnel working on islands and the obtained test values were compared. If the values obtained from testing personnel working on islands were better than those of personnel working on land, it indicated that the LBPQ was more applicable to personnel working on islands. Based on previous reports (24, 25), we focused on seven fit indices: Standardized Root Mean Square Residual (SRMR), χ^2^/d*f* , Comparative Fit Index (CFI), Goodness of Fit Index (GFI), Adjusted Goodness of Fit Index (AGFI), and Root Mean Square Error of Approximation (RMSEA). Generally, a good model data fit is defined as follows: SRMR < 0.08, CFI and AGFI > 0.90, NNFI and GFI > 0.95. The χ^2^/d*f* ratio analyzes the fit of the model by comparing the obtained sample correlation matrix with the estimated correlation matrix under the model. A lower χ^2^/d*f* ratio indicates better fit, reflecting smaller differences between the structure of the observed data and the hypothesized model. Finally, Structural Equation Modeling (SEM) analysis was conducted between the LBPQ and the ODI scale, VAS scale. If the obtained correlation coefficients were good, it indicated that the LBPQ had good fit with classic clinically used scales and was suitable for promotion along with clinically used pain evaluation scales.

## Results

### Participant characteristics and item distribution

**Figure 1:**
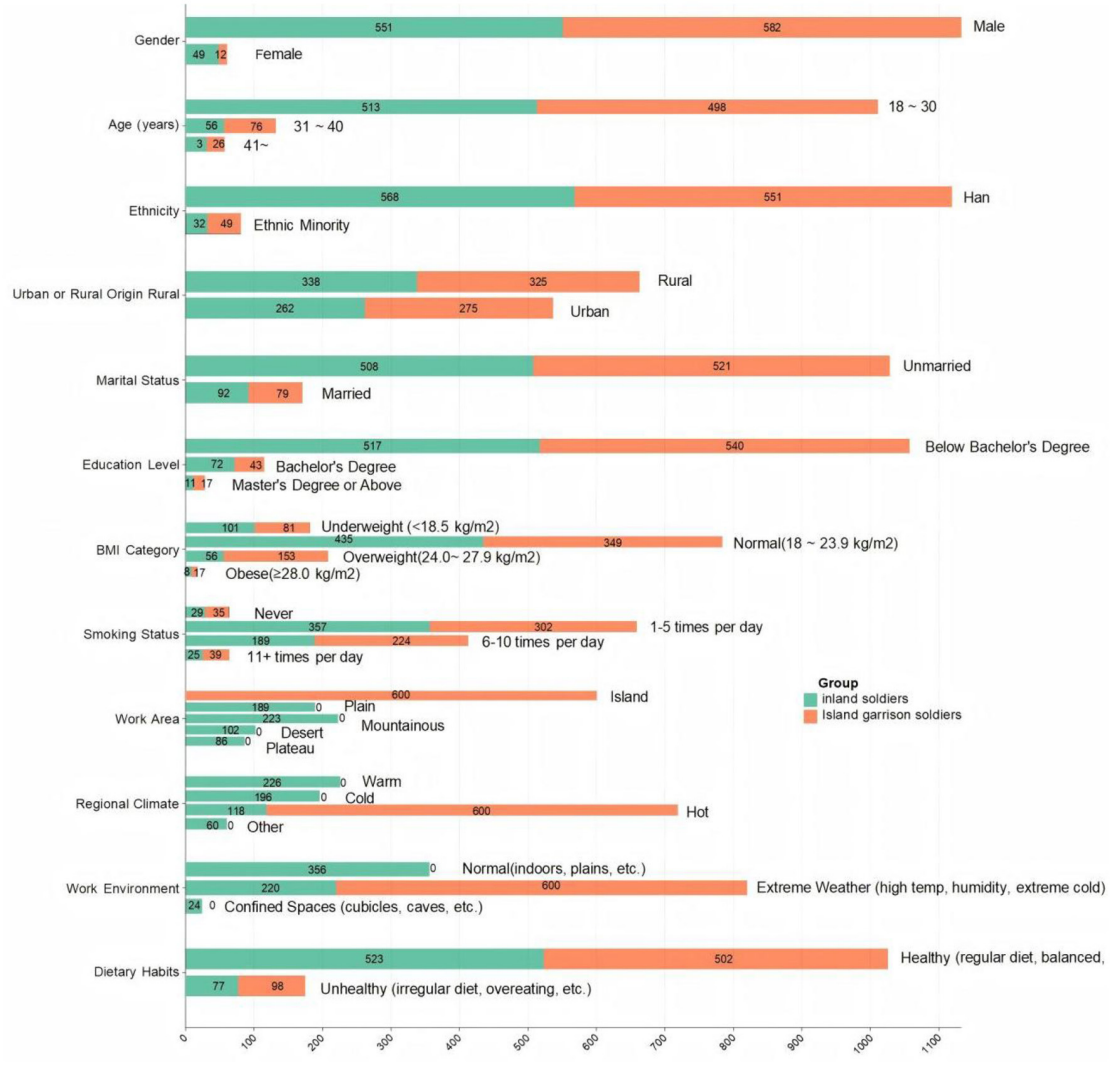
Composition of the surveyed personnel.

A total of 720 personnel working on islands and 750 personnel working on land were tested using the LBPQ. After recovery, 600 valid scales were determined for personnel working on islands, with 120 scales excluded due to missing or multiple selections. Similarly, 600 valid scales were determined for personnel working on land, with 150 scales excluded due to various reasons. The valid number of scales met the sample size requirement (as shown in Figure 1). The mean age of the 600 personnel working on islands was 29.0 ± 6.2 years, with 523 males and 77 females. The mean stationing time on the island was 41.0 ± 9 months. The mean age of the 600 personnel working on land was 26.0 ± 5.1 years, with 462 males and 138 females. The mean stationing time in the unit was 53.0 ± 5.8 months.

### Scale reliability

**Figure 2:**
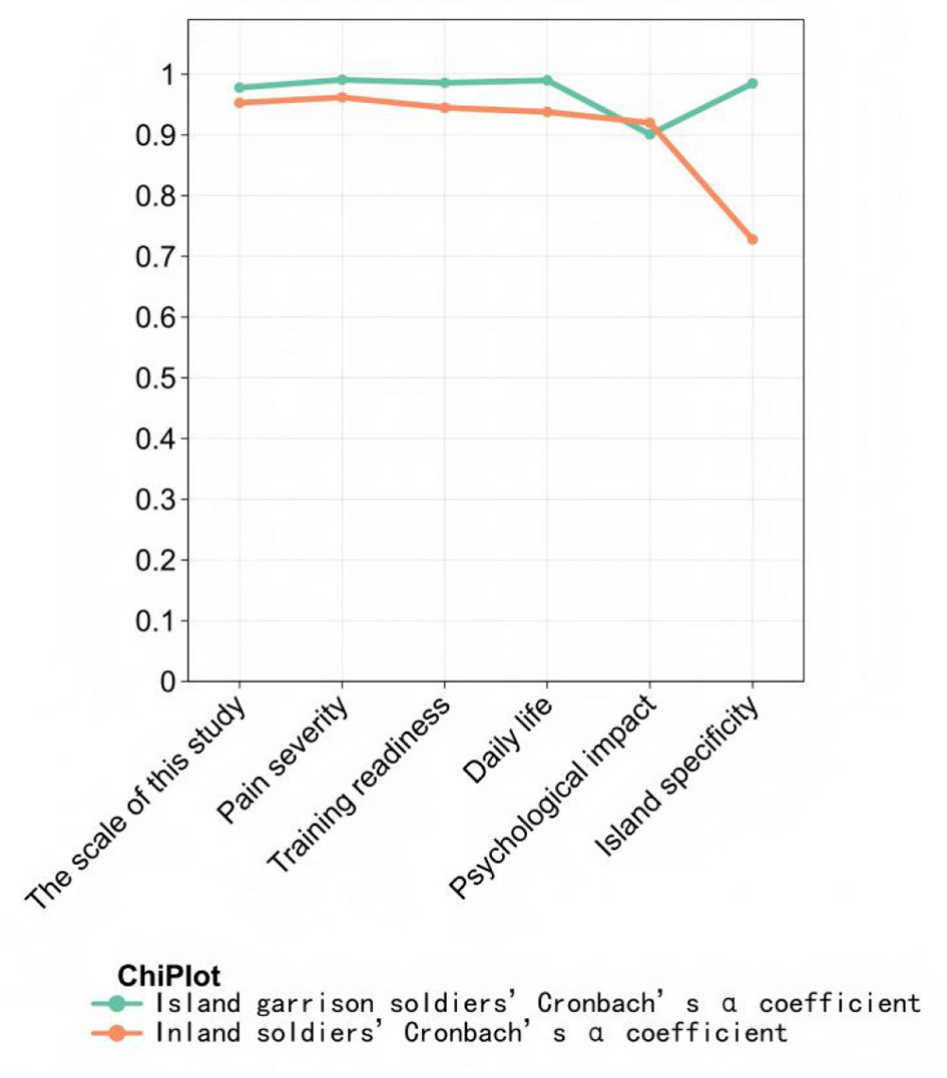
Reliability statistics.

Figure 2 shows scale reliability evaluation. Cronbach's α coefficient of the LBPQ for personnel working on islands was 0.978. The Cronbach's α coefficients for pain severity, training and combat readiness, daily life, psychological impact, and island specificity dimensions were 0.991, 0.986, 0.990, 0.901, and 0.985, respectively. The Cronbach's α coefficient of the LBPQ for personnel working on land was 0.953. The Cronbach's α coefficients for the corresponding dimensions were 0.962, 0.945, 0.938, 0.920, and 0.728, respectively. These results suggest good reliability of the LBPQ and its dimensions, and the overall reliability of the LBPQ is better when applied to personnel working on islands compared to personnel working on land.

### Scale validity analysis

#### KMO test and Bartlett's test of sphericity

The KMO value of the LBPQ for personnel working on islands was 0.967, higher than 0.8. The Bartlett's test of sphericity value was 32,362.202, with *P* < 0.001. Similarly, the KMO value of the LBPQ for personnel working on land was 0.959, higher than 0.8. The Bartlett's test of sphericity value was 14,608.798, with *P* < 0.001. These results indicate that the psychometric properties of both scales are satisfactory and suitable for factor analysis, and the LBPQ is more suitable for factor analysis when applied to personnel working on islands. Using principal component analysis and varimax rotation, five factors with eigenvalues were identified. The cumulative variance contribution rate of the LBPQ was 93.322%, indicating acceptable factors and good validity of the LBPQ for personnel working on islands.

Table 1 shows scale validity evaluation. Items 1, 2, 4, 7, and 19 can be combined into the pain severity dimension; items 3, 6, 8, 16, and 20 into the training dimension; items 9, 11, 13, 14, and 17 into the daily life dimension; items 5, 10, 12, 15, and 18 into the psychological impact dimension; and items 21, 22, 23, 24, and 25 into the island specificity dimension.

**Table 1:** The rotated component matrix.

		**Component**			
**1**		**2**	**3**	**4**	**5**
Item 3	0.870				
Item 8	0.858				
Item 16	0.855				
Item 20	0.855				
Item 6	0.849				
Item 1		0.877			
Item 19		0.872			
Item 7		0.868			
Item 4		0.867			
Item 2		0.863			
Item 13			0.794		
item 14			0.791		
Item 9			0.788		
Item 17			0.783		
Item 11			0.783		
Item 25				0.778	
Item 22				0.775	
Item 21				0.771	
Item 23				0.765	
Item 24				0.751	
Item 15					0.778
Item 18					0.657
Item 5					0.654
Item 12					0.649
Item 10					0.641

Extraction method: Principal Component Analysis. Rotation method: Caesar normalization maximum variance method. Rotation converged after 7 iterations.

#### Convergent validity

**Figure 3:**
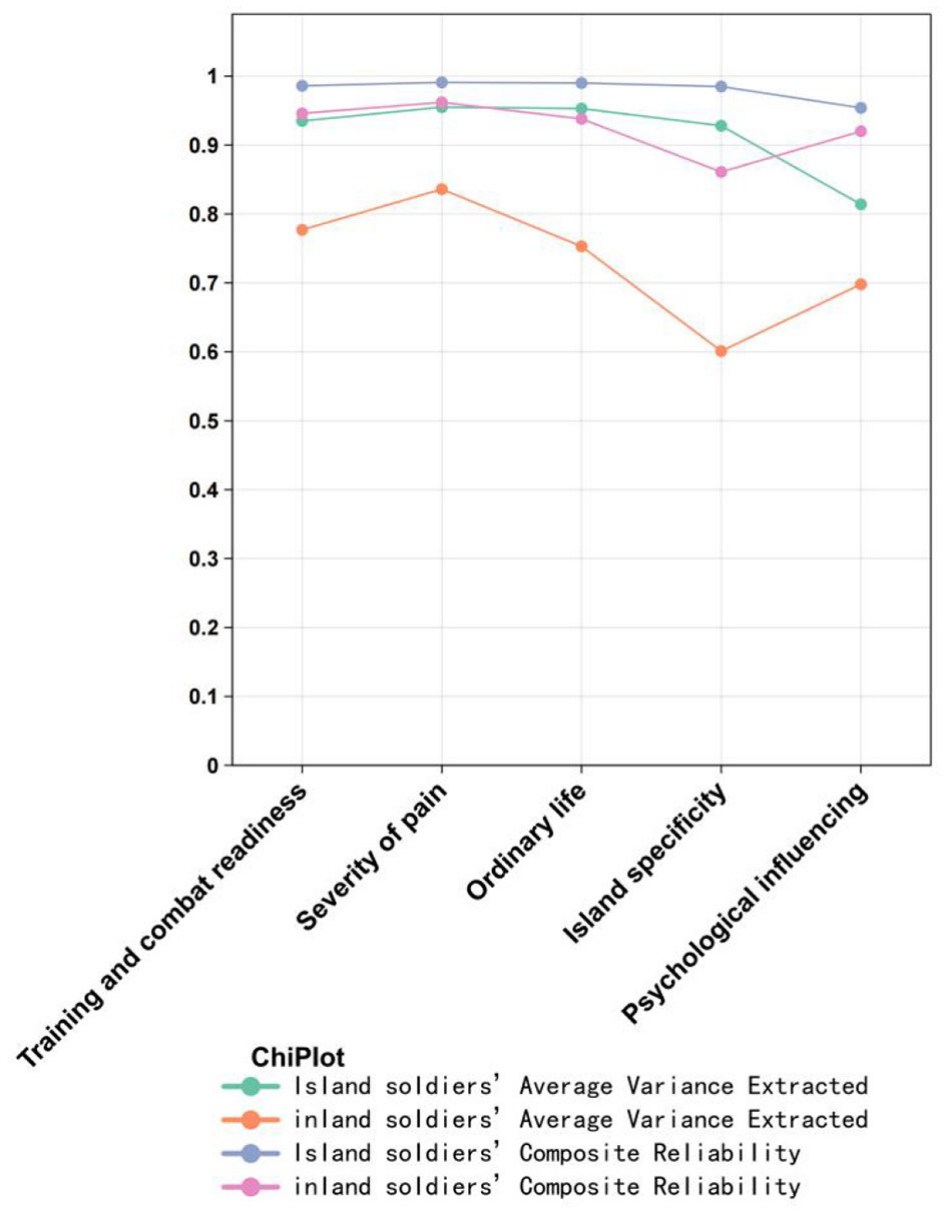
Model AVE and CR indicator results.

Figure 3 demonstrates convergent validity. The AVE of each factor in the LBPQ for both personnel working on islands and land was >0.5, and the CR value was >0.7, indicating good convergent validity of the LBPQ. However, the AVE and CR values of each factor in the personnel working on islands' scale were higher than those in the personnel working on land's group, suggesting higher convergent validity of the LBPQ when applied to personnel working on islands.

#### Discriminant validity

Table 2 shows the analysis of scale discriminant validity. The diagonal line represents the square root of AVE, and the other values represent correlation coefficients. The square root of AVE for each factor in both the personnel working on islands and personnel working on land. LBPQ was greater than the absolute value of the correlation coefficient with other factors, indicating that the LBPQ has discriminant validity for personnel working both on islands and land. Moreover, the square root of AVE and correlation coefficients between factors in the personnel working on islands' scale were higher than those in the personnel working on land's group, suggesting higher correlation and discriminant validity of the LBPQ when applied to personnel working on islands.

**Table 2:** Discriminant validity: Pearson correlation and AVE.

	**Training and combat readiness**	**Severity of pain**	**Ordinary life**	**Island specificity**	**Psychological influencing**
Training and combat readiness	0.967 (0.882)				
Severity of pain	0.570 (0.587)	0.977 (0.914)			
Ordinary life	0.636 (0.598)	0.704 (0.680)	0.976 (0.868)		
Island specificity	0.701 (0.324)	0.688 (0.270)	0.773 (0.296)	0.963 (0.775)	
Psychological influencing	0.687 (0.656)	0.561 (0.570)	0.731 (0.726)	0.674 (0.299)	0.902 (0.835)

The diagonal line numbers represent the square root of AVE.

Outside the parentheses is data of personnel working on islands, inside the parentheses is data of personnel working on land.

#### Model fit indices

Table 3 shows model fit evaluation. χ^2^/d*f* is the core index of model fitting. When χ^2^/d*f* < 3, it indicates that the model fitting is good. All confirmatory factor analysis model fit indicators for the LBPQ for personnel working on islands meet the standards, with χ^2^/d*f* = 1.354 (< 3), Comparative Fit Index (CFI) = 0.997 (>0.9), Root Mean Square Error of Approximation (RMSEA) = 0.024 (< 0.10), Goodness of Fit Index (GFI) = 0.954 (>0.9), and Root Mean Square Residual (RMR) = 0.008 (< 0.05). However, some of the confirmatory factor analysis model fit indicators for the LBPQ among personnel working on land do not meet the standards, with χ^2^/d*f* = 3.033 (< 3), and Root Mean Square Residual (RMR) = 0.096 (< 0.05). These results indicates that the model fits better for personnel working on islands.

**Table 3:** Model fitting indicators.

**Common indicators**	**χ^2^**	**d*f***	***p***	**χ^2^/d*f***	**GFI**	**RMSEA**	**RMR**	**CFI**	**NFI**	**NNFI**
Judgment criteria	–	–	>0.05	< 3	>0.9	< 0.10	< 0.05	>0.9	>0.9	>0.9
Value	Island personnel 358.818 Land personnel (803.671)	265	0.000	1.354 (3.033)	0.954 (0.918)	0.024 (0.058)	0.008 (0.096)	0.997 (0.963)	0.989 (0.946)	0.997 (0.958)

Default Model: $\chi_{\left(300\right)}^{2}$ = 32,920.013, *p* = 1.000.

#### Correlation analysis

Table 4 shows the covariance relationships of the scale. Using Structural Equation Modeling (SEM) to analyze the correlations between LBPQ and ODI, VAS through covariance calculations, we obtained the covariance relationships (i.e., correlations) between the scales. The standard estimated coefficient (i.e., correlation coefficient) between LBPQ and ODI was found to be 0.824 with a standard error of 0.023. The standard estimated coefficient (i.e., correlation coefficient) between LBPQ and VAS was 0.675 with a standard error of 0.04. This indicates that when the standard error is < 0.05, the correlation coefficients between LBPQ and both ODI and VAS are >0.6, suggesting that the LBPQ scale has a good correlation with both the VAS and ODI scales.

**Table 4:** Covariance matrix.

***X***	***Y***	**Standard error**	***z***	***p***	**Standard estimate**
LBPQ	ODI	0.023	24.470	< 0.01	0.824
LBPQ	VAS	0.040	20.215	< 0.01	0.675

## Discussion

In this instrument development study, the results revealed that the LBPQ presented better convergent and discriminant validity in the islands' group than in the land group, indicating that the newly developed tool was specifically designed and suitable for personnel working on islands. Although several similar questionnaires have been developed previously to evaluate LBP, none was designed to assess personnel working on islands. Accurately measuring the status of LBP is essential to give healthcare providers better advice for improving personnel health. Personnel working on islands is a special group. Daily intensive training and the 4-high environment make the conventional scales unsuitable for evaluating LBP. Thus, in the present study, we successfully developed a 25-item assessment tool for evaluating LBP in personnel working on islands. To keep fit with the characteristics of the personnel working on islands, we divided the questionnaire into 5 dimensions associated with LBP: pain intensity and frequency, training, daily life, mental effects and island characteristics. The results of PCA also support the 5-dimension design of LBPQ.

Study showed that evaluating physical and mental state in a timely and accurate manner would help enhance the working ability of personnel, especially in an enclosed environment such as small islands (26). Hence, several specific questionnaires were used to assess comprehensive health status for personnel working on islands, including the Comprehensive Soldier & Family Fitness (CSF2) (27), the Military to Civilian Questionnaire (M2C-Q) (28), and the Global Assessment Tool (GAT) (29). However, these questionnaires were developed and used in the USA and other western countries. Also, these tools were designed with different structures and applicability for targeting personnel with different requirements (30). Furthermore, the training contents for personnel working on islands are quite different from those in other countries. Considering the small scope of activities and enclosed environment on islands, mental disorder caused by LBP would also become a common problem on islands. Thus, the items associated with pain intensity and frequency and mental effects were also completed under the consultation of psychologists.

The results revealed that the fitness of LBPQ for personnel working on islands is better than that for personnel working on land. The explanation may be that some of the items were closely associated with the life and environment on islands. The temperature and humidity might be similar with the hometown of some personnel. However, most personnel have never been to islands before joining the army and the environment is completely new and different.

Besides specific types of LBP, several specific factors lead to the increasing incidence rate of LBP. One major problem is drinking water. The water on the islands is recollected from rain and filtered 2-3 times before drinking. However, impurities are still a problem leading to increasing incidence of calcium crystallization or even development of kidney stones. And sweating profusely after working the island operation under such a hot environment without intake of enough water may also result in renal crystallization or stone formation in some individuals. Another common factor associated with LBP was bites by red fire ants (Solenopsis Invicta). Patients bitten by these insects experience pain and itching around the wound site, and some patients even progress to having systemic symptoms.

The result revealed that the pain intensity and frequency dimension correlated excellently with VAS, which was in accordance with our hypothesis. It indicates that the LBPQ has good accuracy in measuring pain levels, providing assessors with intuitive feedback to quickly evaluate the pain levels of personnel working on islands (29). LBPQ also correlated well with the ODI scale, even better than its correlation with the VAS pain scale. ODI is widely used in clinical practice to assess the degree of functional limitation caused by LBP, helping clinicians understand patients' pain levels more accurately and the impact on daily life. The excellent correlation between LBPQ and ODI demonstrates that the LBPQ has a high assessment effect in evaluating LBP functional impairment, and it has good specificity and feasibility in assessing the pain levels of personnel working on islands. This lays a solid theoretical foundation for the subsequent use of LBPQ in monitoring and evaluating the LBP of personnel working on islands.

## Limitations

The present instrument development study has several limitations. First, LBPQ contains 25 items, requiring 8–10 min to complete. Shortening it to 3–5 min would be better. Second, the specificity and compatibility of LBPQ for personnel working on different islands need to be examined further because certain differences in climate, living environment, food supply, and other aspects between different islands necessitates validation of the applicability of the LBPQ on more islands. Third, further evaluation of the LBPQ is necessary to correlate it with more LBP assessment scales and tools used in clinical and research settings to verify its applicability and reliability in assessing LBP. Fourth, since it is necessary to select personnel working on islands with LBP to independently judge and fill in questions related to subjective feelings such as pain intensity and psychological impact in the LBPQ, there may be selection bias and information bias. Fifth, if there are plans to adapt the LBPQ to people in other regions or ethnic groups in the future, differences in cultural backgrounds and language habits may become key influencing factors, requiring cultural adaptation and re-validation. Additionally, the assessment content of the LBPQ should be refined to enable a more comprehensive evaluation of the pain factors for personnel working on islands.

## Conclusions

The LBPQ is the first scale of its kind developed for personnel working on islands. Results of the present study confirm that LBPQ has good reliability and validity and can be used specifically for personnel working on islands to evaluate their status of LBP. Further study would be conducted to investigate more extensive usage of LBPQ in different special environments.

## Data Availability

The data analyzed in this study is subject to the following licenses/restrictions: All data produced or examined throughout this study are incorporated within this published article. Only the results of data analysis can be published publicly. Requests to access these datasets should be directed to XZ, 13818909826@163.com.
